# Engineered Human Heavy-Chain Ferritin with Half-Life Extension and Tumor Targeting by PAS and RGDK Peptide Functionalization

**DOI:** 10.3390/pharmaceutics13040521

**Published:** 2021-04-09

**Authors:** Shuang Yin, Yan Wang, Bingyang Zhang, Yiran Qu, Yongdong Liu, Sheng Dai, Yao Zhang, Yingli Wang, Jingxiu Bi

**Affiliations:** 1 School of Chemical Engineering and Advanced Materials, The University of Adelaide, Adelaide SA5005, Australia; shuang.yin@adelaide.edu.au (S.Y.); bingyang.zhang@adelaide.edu.au (B.Z.); yiran.qu@adelaide.edu.au (Y.Q.); 2 School of Chinese Medicine and Food Engineering, Shanxi University of Traditional Chinese Medicine, Jinzhong 030619, China; wangyan81823@aliyun.com; 3 State Key Laboratory of Biochemistry Engineering, Institute of Process Engineering, Chinese Academy of Sciences, Beijing 100190, China; ydliu@ipe.ac.cn (Y.L.); zhangyao@ipe.ac.cn (Y.Z.); 4 Department of Chemical Engineering, Brunel University London, Uxbridge UB8 3PH, UK; sheng.dai@brunel.ac.uk

**Keywords:** ferritin, drug delivery, tumor targeting, half-life extension

## Abstract

Ferritin, one of the most investigated protein nanocages, is considered as a promising drug carrier because of its advantageous stability and safety. However, its short half-life and undesirable tumor targeting ability has limited its usage in tumor treatment. In this work, two types of functional peptides, half-life extension peptide PAS, and tumor targeting peptide RGDK (Arg-Gly-Asp-Lys), are inserted to human heavy-chain ferritin (HFn) at C-terminal through flexible linkers with two distinct enzyme cleavable sites. Structural characterizations show both HFn and engineered HFns can assemble into nanoparticles but with different apparent hydrodynamic volumes and molecular weights. RGDK peptide enhanced the internalization efficiency of HFn and showed a significant increase of growth inhibition against 4T1 cell line in vitro. Pharmacokinetic study in vivo demonstrates PAS peptides extended ferritin half-life about 4.9 times in Sprague Dawley rats. RGDK peptides greatly enhanced drug accumulation in the tumor site rather than in other organs in biodistribution analysis. Drug loaded PAS-RGDK functionalized HFns curbed tumor growth with significantly greater efficacies in comparison with drug loaded HFn.

## 1. Introduction

Ferritin is one of the most attractive protein nanocages for drug delivery, due to its extraordinary thermal and chemical stability. In mammals, ferritin is a 12 nm sphere with an 8 nm cavity, made up of 24 subunits [1]. Two types of ferritin subunits exist in mammal tissues, called heavy-chain (H-chain) and light-chain (L-chain) (21 kDa and 19 kDa), respectively. Both two types of subunits consist of five α-helices (helices A-E), one long loop connecting helix B and C (BC loop) and three turns connecting helices. Exposed BC loop of Human H-chain ferritin (HFn) has a binding site of human transferrin receptor 1 (TfR1) and gives rise to an intrinsic tumor active targeting ability [2]. Researchers have loaded various chemotherapeutics into H-chain ferritin and explored its anti-tumor efficacy. For example, 5-fluorouracil attached Au nanoparticles inside ferritin decreased IC_50_ against HepG2 cells by 15 times [3]. A single dose of doxorubicin (DOX) loaded HFn (HFn/DOX) successfully inhibited TfR1 overexpressed HT-29 human colon cancer cells growth in mice [4]. Neuronal drugs carbachol and atropine loaded ferritin is proven to be able to regulate pancreatic cancer progression [5].

In spite of ferritin’s multiple advantages, it is still facing challenges as a drug nanocarrier. It has a half-life in circulation of approximate 2 h in rats, shorter than the majority of other drug nanocarriers because of its relatively small particle size. Wang fused albumin binding domain (ABD) to increase ferritin half-life to 17.2 h [6]. In addition, the innate tumor targeting ability of HFn cannot be guaranteed in all tumors. The expression level of its receptor, human TfR1, varies in different tumor cell lines and in different stages of tumor progression [7,8]. Head and neck cancer, colorectal cancer and cervical cancer tissues have the highest expression level of human TfR1, whilst no human TfR1 was detected in carcinoid, prostate and testicular tumor tissues [9]. Human TfR1 is also ubiquitously expressed in healthy human tissues, such as bone marrow, lung, colon and liver, to import iron into cells, so the usage of HFn has the risk of undesired drug accumulation in healthy tissues. 

To address (1) the short half-life and (2) the limited tumor targeting ability of HFn, two functional peptides were fused to HFn subunit C-terminal to construct three functionalized HFns (HFn-PAS, HFn-GFLG-PAS-RGDK and HFn-PLGLAG-PAS-RGDK). One peptide, PAS peptide, comprises repetitive P, A and S residues. It was designed by Schlapschy, and aimed to mimic poly ethylene glycol (PEG) [10]. In three previous studies, Falvo et al. have fused 40 aa and 75 aa PAS peptides to human ferritin subunit at N-terminal to increase half-life in circulation [11,12,13]. Another peptide is a tetrapeptide named as RGDK. It belongs to tumor penetration peptide (TPP) and possesses two functions. RGDK enhances drug tumor delivery, and drug distribution inside whole tumor tissue instead of only tumor cells alongside tumor vessels [14]. It specifically binds to two receptors, integrin αvβ3/5 and neuropilin-1, both overexpressed in a wide range of tumor cells [15]. Integrin αvβ5 is highly expressed in cancers such as gliomas and urothelial cancer, and neoropilin-1 expression is upregulated in ovarian cancer, colorectal cancer and stomach cancer [9]. Therefore, the addition of RGDK peptide can improve HFn tumor targeting ability and broaden HFn application. The GFLG (Gly-Phe-Leu-Gly) and PLGLAG (Pro-Leu-Gly-leu-Ala-Gly) in HFn-GFLG-PAS-RGDK and HFn-PLGLAG-PAS-RGDK are enzyme cleavable sites responding to cathepsin B and matrix metalloproteinase-2/9 (MMP-2, MMP-9), respectively [16,17]. Both enzymes are overexpressed in tumors but Cathepsin B is located inside cell lysosome and MMP-2 is secreted outside tumor cells [18,19]. The PAS-RGDK functional moiety in these two dually-functionalized HFns is theoretically to be cleaved from HFn before and after cell internalization, respectively. In total, four HFn-based proteins were compared with each other to investigate the impacts of PAS and RGDK on HFn performance as a drug nanocarrier. 

A total of four HFn-based proteins were expressed in *Escherichia coli* (*E. coli*), and purified. High-Performance Size Exclusion Chromatography coupled with Multiple Angle Laser Light Scattering (HPSEC-MALLS) was used to characterize protein structures. In vitro and in vivo tests were designed to compare anti-tumor drug delivery performance of four HFn-based proteins in tumors lacking overexpressed human TfR1. Therefore, 4T1, a BALB/c mice breast tumor cell line was selected; 4T1 does not express human TfR1 and overexpresses integrin αvβ3/5 and neuropilin-1 [20,21]. Cellular uptake assay investigated RGDK functionalization impact on 4T1 cellular internalization efficiency. Intracellular distribution monitored if drug can be released from proteins and enter nucleus for killing tumor cells after internalization. Cytotoxicity assay compared IC_50_ values of drug carried by four HFn-based proteins. Pharmacokinetic study mainly assessed PAS impact on half-life in circulation. Biodistribution study assessed tumor targeting ability of four HFn-based proteins. In vivo anti-tumor test was conducted to compare the tumor growth inhibition efficacy of DOX carried HFn and functionalized HFns. 

## 2. Materials and Methods

### 2.1. Materials

A total of four recombinant HFn-based proteins (HFn, HFn-PAS, HFn-GFLG-PAS-RGDK and HFn-PLGLAG-PAS-RGDK) were expressed in *Escherichia coli* (*E. coli*) BL21 (DE3). HFn-PAS was constructed by inserting a 15 aa flexible linker (GGGSGGGTGGGSGGG), an enzyme-cleavable site GFLG, a 40 aa PAS peptide (ASPAAPAPAPAAPAPSAPAASPAAPAPASPAAPAPSAPA) together with another 5 aa flexible liner (GGSGG) to HFn Subunit C-terminus. HFn-GFLG-PAS-RGDK was constructed by adding RGDK tretapeptide to HFn-PAS C-terminus. HFn-PLGLAG-PAS-RGDK, was designed by substitution of enzyme-cleavable site GFLG in HFn-GFLG-PAS-RGDK by a six residue MMP-2 cleavable site PLGLAG. Proteins were purified using a two-step pathway. Briefly, HFn was purified by heat-acidic precipitation at 60 °C, pH 4.5 5 min followed by butyl fast flow hydrophobic interaction chromatography (GE Healthcare, Waukesha, WI, USA). The other three functionalized HFns were purified by heat-acidic precipitation at 60 °C, pH 4.5 5 min followed by mono Q ion-exchange chromatography (GE Healthcare, Waukesha, WI, USA).

Doxorubicin hydrochloride (DOX) was purchased from Dalian Meilun Biotechnology (Dalian, China). 4T1 cells were purchased from Cellbank (Sydney, NSW, Australia). RPMI-1640 medium, penicillin-streptomycin solution (100 ×), fetal bovine serum (FBS), 0.25% trypsin-EDTA (1 ×) solution, Hoechst 33258 reagent and MTT reagent were purchased from Invitrogen (Thermo Scientific, Adelaide, SA, Australia). Propidium iodide and trypan blue solution were bought from Sigma-Aldrich (Sydney, NSW, Australia). All of the other reagents were of analytical reagent quality. Mili Q water was utilized throughout the whole procedure, produced by Merck Mili Q direct (Melbourne, VIC, Australia).

### 2.2. HPSEC-MALLS Characterization of Purified Proteins and DOX Loading

The four protein purities were analyzed by reducing 12% SDS-PAGE (Bio-Rad, Gladesville, NSW, Australia). Sizes and molecular weights (Mws) were measured by HPSEC-MALLS. In HPSEC-MALLS analysis, TSK G4000 SWxl column (Tosoh bioscience, Tokyo, Japan) was connected to HPLC (Shimadzu, Melbourne, VIC, Australia) coupled with DAWN MALLS and Optilab refractive index (RI) detector (Wyatt, Santa Barbara, CA, USA). Equilibration buffer was 20 mM phosphate buffer (PB), 0.1 M Na_2_SO_4_, pH 7.0. Flow rate was 0.8 mL min^−1^. Absorbance of fractions at 280 nm was monitored. Sample loading volume was 50 µL.

In DOX loading, briefly, 1 mg mL^−1^ HFn-based protein in 20 mM phosphate buffer, 5 mM guanidinium chloride, pH 7.5 was heated at 50 °C for 6 h with 0.2 mg mL^−1^ DOX. Excessive DOX was separated from DOX loaded protein (protein/DOX) by desalting on Hitrap G25 desalting column (GE Healthcare, Waukesha, WI, USA) using AKTA PURE (GE Healthcare, Waukesha, WI, USA). Collected protein/DOX underwent measurement of OD280 and OD480. DOX has absorbance at both 280 and 480 nm, and protein only has absorbance at 280 nm. Therefore, two (2) assumptions were made: (1) OD480_protein/DOX_ = OD480_DOX_; (2) OD280_protein/DOX_ = OD280_DOX_ + OD280_protein_. Standard OD vs. C linear curves of DOX and HFn-based proteins were determined by serial concentrations of DOX (1–40 μg mL^−1^) and proteins (0.1–1.2 mg mL^−1^). Standard curves were used to calculate the concentration of DOX (C_DOX_) and the concentration of proteins (C_protein_) in protein/DOX. Consequently, the calculation of loading ratio in protein/DOX was as follows:

(1) $$\mathrm{Loading}\;\mathrm{ratio}\;=\frac{\mathrm{number}\;\mathrm{of}\;\mathrm{DOX}}{\mathrm{number}\;\mathrm{of}\;\mathrm{protein}}\;=\;\frac{C_{\mathrm{DOX}}{•\mathrm{Mw}}_{\mathrm{protein}}}{C_{\mathrm{protein}}{•\mathrm{Mw}}_{\mathrm{DOX}}}$$

### 2.3. Cellular Uptake Test

The 4T1 cell line was cultured in RPMI-1640 medium supplemented with 10% FBS at 37 °C in a 5% CO_2_ atmosphere. Cellular uptake test procedure was modified from a previous paper [15]. For each protein/DOX group, three different treatments were conducted to obtain three fluorescence intensities, total fluorescence, internalized fluorescence, fluorescence after RGDK peptide pre-incubation.

The procedure was as follows: (1) Cell seeding. 4T1 Cells in the exponential growth phase were seeded in 24-well plates at a density of 1 × 10^5^ cells per well and cultured for 48 h for attachment. (2) RGDK peptide pre-incubation in wells for fluorescence after RGDK peptide pre-incubation determination. To investigate the impact of fused RGDK on cellular uptake characteristics, 500 μM free RGDK peptide was pre-incubated with the cell for 1 h at 37 °C to saturate RGDK specific receptors. (3) Drug incubation. The media with or without RGDK peptide in all wells were discarded and cells were washed with phosphate buffered saline (PBS) three times, prior to adding 100 μL serum-free culture medium containing free DOX or protein/DOX (15 μg mL^−1^ DOX-equivalent). Then the cells were incubated for 90 min at 37 °C and washed three times with PBS to remove drugs. (4) Trypan blue quenching. In wells for internalized fluorescence and fluorescence after RGDK peptide pre-incubation determination in all five groups, cells were incubated with trypan blue (0.25% in 0.85% NaCl) for 5 min at 25 °C, and then washed five times with PBS to remove trypan blue. (5) Detachment of cells for flow cytometry analysis. A total of 400 μL of 0.25% trypsin–0.05% EDTA solution was added to all wells for digestion for 5 min at 37 °C and 2 mL of complete medium was added to stop the digestion. Detached cells were spun at 112 rcf for 3 min at 4 °C and re-suspended in 1 mL PBS. In total, five microliters of propidium iodide (PI) was added to incubate with cells for 10 min at 25 °C for differentiation of alive and dead cells in flow cytometry detection. (6) Flow cytometry analysis. Csampler flow cytometry (Becton Dickinson, San Jose, CA, USA) was employed to determine the mean fluorescence of 5000 cells in each sample. A cell control underwent PI staining but without drug incubation, trypan blue and RGDK peptide treatment was used for gating and parameter setting prior to sample detection. PE channel (excitation laser light: 488 nm, emission: 578 nm) was utilized for DOX fluorescence detection. Mean fluorescence intensity of each sample was recorded. 

### 2.4. Intracellular Distribution Analysis

Intracellular distribution analysis was designed to monitor if DOX carried by HFn-based proteins could reach tumor cell nucleus for disruption of cell division. Exponentially growing 4T1 cells were placed on a 6-well plate at a density of 4 × 10^5^ cells per well and cultured for 24 h. One cover-glass slide was put in each well prior to seeding. The medium was then discarded and cells were treated with fresh media containing protein/DOX or free DOX (20 μM DOX-equivalent) in 2 mL per well for 3 h. Drugs in wells were then removed and cells were washed three times using PBS. Fresh complete medium was added to wells for another 36 h incubation. Subsequently, the cells were washed three times with PBS and fixed with 4% paraformaldehyde for 10 min at 25 °C. Following another three times wash with PBS, cell nucleus were stained with 0.5 μg mL^−1^ Hoechst 33258 at 25 °C for 5 min. A ZOE fluorescence cell imager (Bio-Rad, Gladesville, NSW, Australia) was used to visualize cells. Images of cells under bright filed channel, green channel (Excitation: 480/17 nm, Emission: 517/23 nm) and blue channel (Excitation: 355/40 nm, Emission: 433/36 nm) were captured. Green channel and green channel monitored Hoechst 33258 and DOX signal, respectively.

### 2.5. Cytotoxicity Study

The cytotoxicity of four protein/DOX and free DOX against 4T1 cell was evaluated by MTT assays. Exponential growth-phase cells were digested by 0.25% trypsin-0.05% EDTA, and cell density was adjusted to 1 × 10^5^ cells per mL by complete medium. 100 μL of cells were seeded in wells of 96-well plates. Then, four wells without cells were adopted as blank control on each plate. After incubation for 24 h, medium was replaced with new complete medium separately containing either free DOX or protein/DOX, whose concentrations ranged from 0 to 30 μg mL^−1^ (equivalent DOX). Four 0 μg mL^−1^ DOX wells on each plate were cell control wells. After incubation for another 60 h, the media were removed and cells were washed three times by PBS. Then, 90 μL of new complete medium with 10 μL of MTT solution was added to each well for another 4 h. A total of 100 μL dimethyl sulfoxide (DMSO) was added to wells to ensure complete solubilization of the formed form-azan crystals. Finally, the absorbance of the solution was measured at 595 nm (background: 630 nm) by a Microplate Reader (Biotek, Winooski, VT, USA). Absorbance of each well (A_well_) was defined as A_595_–A_630_. Cell viability (%) were calculated using Equation (2). A_cell_ was the A_well_ of cell controls, and A_blank_ was the A_well_ of blank controls. IC_50_ value of each group was calculated using dose-response fitting in origin 9.0 software (Originlab, Northampton, MA, USA).

Cell viability (%) = (A_well_ − A_blank_)/(A_cell_ − A_blank_) × 100 (%) (2)

### 2.6. Pharmacokinetics Study

All animal experiments were performed with the approval of the medical ethics committee of Shanxi University of Chinese Medicine (Approval Number 2019LL137, approval date: 13 June 2019). Specific-pathogen free Sprague Dawley rats (male, 230–250 g, SPF Biotechnology Co., Ltd. Beijing, China) were randomly assigned to six groups (three rats in each group), and administrated with PBS, free DOX and protein/DOX (3.0 mg kg^−1^ DOX equivalent) separately via intravenous injection at tail vein. After injection, blood samples were collected from the retro orbital sinus at fixed time points (10, 30 min, 1, 2, 4, 8, 12, 24, 36, 48 h) and followed by clotting for at least 0.5 h at 37 °C. Serum was obtained by centrifugation at 4032 rcf for 30 min at 4 °C. Finally, 100 μL of serum of each sample was transferred to a 96-well microplate, and the DOX contents were determined using SpectraMax i3x microplate reader (Molecular devices, San Jose, CA USA). Excitation wavelength was set at 480 nm and emission at 580 nm. Meanwhile, the standard curve of the fluorescence intensity with varying concentrations of DOX in rat serum was also measured for quantitative analysis. Half-lives of DOX and protein/DOX were calculated using Drug Analysis System 2.0 software (Drug China, Shanghai, China) by fitting data in single-compartment mode.

### 2.7. In Vivo Imaging

The four HFn-based proteins were first labelled by Sulfo-cy5 NHS ester (Lumiprobe, Hunt Valley, MD, USA) with a molar ratio of 1:30 (Protein to Cy5) and the uncoupled Cy5 was removed by Hitrap G25 desalting chromatography. As 4T1 is a BALB/c breast tumor cell line, female BALB/c mice were chosen to establish tumor-bearing animal model. 1 × 10^6^ 4T1 cells in 100 μL of PBS were injected into right armpit of 8-week old female BALB/c mice (specific-pathogen free, SPF Biotechnology Co., Ltd. Beijing, China) to form mice tumor model. Each group had three mice. When tumor volume reached about 300 mm^3^, a 150 μL sample of Cy5 or protein-Cy5 conjugates (0.2 mg kg^−1^ Cy5 equivalent) was intravenously injected into the tumor-bearing mice via tail vein. After treatment, the mice were anesthetized using isoflurane at 2, 4, 6.5, 12, 24 and 52 h and fluorescence images were taken under excitation wavelength of 646 nm and emission wavelength of 662 nm using FX Pro in vivo imaging system (Bruker BioSpin, Carteret, NJ, USA). 

### 2.8. Anti-Tumor Assay

Then, 1 × 10^6^ 4T1 cells in 100 μL of PBS were injected into right armpit of 8-week old female BALB/c mice. For in vivo inhibition of tumor progression assessment, female BALB/c mice bearing 4T1 tumors of approximate 250 mm^⁠3^ in size were randomly assigned to six groups (*n* = 6 in each group) and treated with protein/DOX (3 mg kg^−1^ DOX equivalent), free DOX (3 mg kg^−1^), or PBS via 200 μL intravenous injection. The drug injection was carried out every 4 days for two doses. The volumes of tumors were measured every other day. Mice were monitored for up to 17 days post-implantation and then sacrificed. Primary tumors were harvested for ex vivo imaging. 

### 2.9. Statistical Analysis

Data were presented in Mean ± Standard deviation (SD). T-test was applied to evaluate statistical significance of results. *p* value < 0.05 was considered significant.

## 3. Results

### 3.1. Purified HFn-Based Protein Characterizations and Drug Loading 

**Figure 1:**
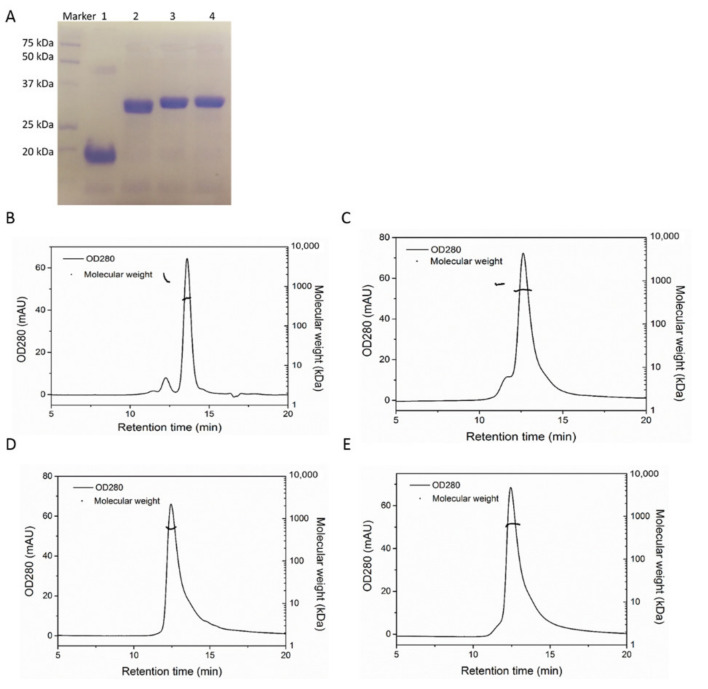
Characterizations of purified HFn-based proteins. (**A**), 12% reducing SDS-PAGE analysis of purified proteins. Lane 1, HFn; 2, HFn-PAS; 3, HFn-GFLG-PAS-RGDK; 4, HFn-PLGLAG-PAS-RGDK. (**B**), HPSEC-MALLS chromatogram of HFn. (**C**), HPSEC-MALLS chromatogram of HFn-PAS. (**D**), HPSEC-MALLS chromatogram of HFn-GFLG-PAS-RGDK. (**E**), HPSEC-MALLS chromatogram of HFn-PLGLAG-PAS-RGDK.

The purity of each protein after purification reached above 90% based on the SDS-PAGE gel (Figure 1A), calculated by density scan using software Image J [22]. The apparent subunit molecular weights of HFn-PAS, HFn-GFLG-PAS-RGDK and HFn-PLGLAG-PAS-RGDK on gel were higher than their theoretical molecular weights (26 kDa, 26.5 kDa and 26.6 kDa), which are due to the hydration of PAS peptides. Bands of two PAS-RGDK functionalized HFns in SDS-PAGE gel were slightly higher than HFn-PAS probably due to the presence of extra residues. 

The apparent hydrodynamic radius and molecular weight (Mw) of four HFn-based proteins were further characterized by HPSEC-MALLS analysis. In Figure 1B–E, the horizontal Mw lines of the main peaks show the uniform Mws of all four HFn-based proteins. Table 1 lists the hydrodynamic size and Mw of each protein. Due to PAS peptides, HFn-PAS possessed 1.4 nm higher apparent hydrodynamic radius in contrast to HFn. The adding of RGDK peptide and enzyme-cleavable site into HFn slightly further increased hydrodynamic radius. HFn-GFLG-PAS-RGDK and HFn-PLGLAG-PAS-RGDK were 1.75 nm and 1.91 nm larger than HFn, respectively. MALLS determined Mw order is in accordance with theoretical order: HFn-PLGLAG-PAS-RGDK > HFn-GFLG-PAS-RGDK > HFn-PAS > HFn, and average Mw of all three proteins determined are similar to their theoretical Mw (Table 1). 

After purification, the model drug DOX was loaded by thermally induced passive diffusion. On average, incubation with DOX at 50 °C loaded 33.5, 38.4, 36.9 and 42.1 DOX in one HFn, HFn-PAS, HFn-PLGLAG-PAS-RGDK and HFn-GFLG-PAS-RGDK nanocage, respectively. HFn DOX loading ratio in this study is comparable with previous pH-induced disassembly–reassembly method [23] and 8 M urea method adopting HFn [4].

### 3.2. Cellular Uptake Efficiency

**Figure 2:**
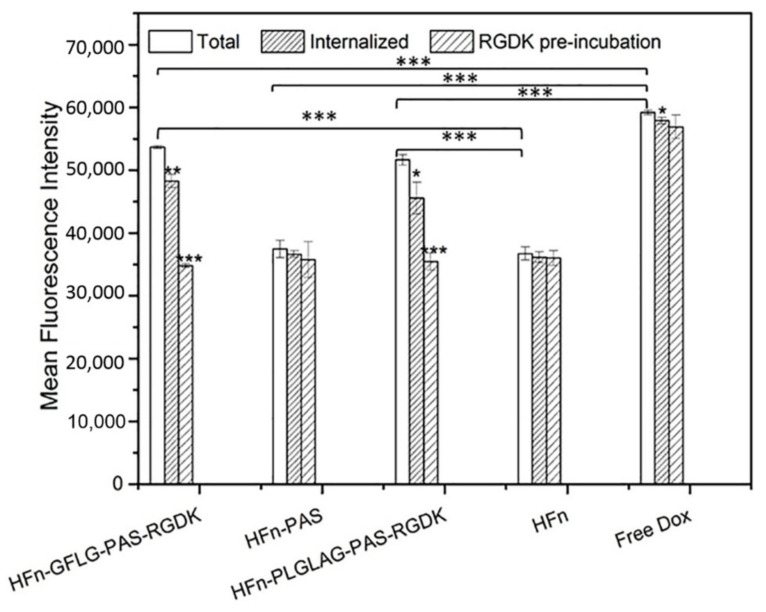
Mean DOX fluorescence intensity in 4T1 cellular uptake test. Data were represented as mean ± standard deviation (*n* = 3), * *p* < 0.05, ** *p* < 0.01, *** *p* < 0.001. Symbol ‘*’ on top of column represents the significance of *p* value between this column and the white column (total DOX fluorescence) in the same group.

In cellular uptake test, we investigated the RGDK functionalization impact on cellular uptake efficiency and the mechanism. Figure 2 presents DOX fluorescence intensities of all groups. Total fluorescence of DOX measured in flow cytometry came from two sources, DOX internalized by 4T1 cells and un-specifically bound to cell membranes. Trypan blue treatment quenched the signal from membrane-bound DOX, and, therefore, a lower internalized fluorescence intensity compared with total fluorescence intensity was observed in all groups (Figure 2). 

Free DOX showed significantly greater internalized cellular uptake than others. HFn-GFLG-PAS-RGDK/DOX and HFn-PLGLAG-PAS-RGDK/DOX had the second highest efficiencies and were significantly different from the rest two. This means the insertion of RGDK peptide has significantly enhanced cellular uptake efficiency. HFn-PAS/DOX and HFn/DOX had similar internalized cellular uptake efficiencies. RGDK peptide pre-incubation treatment, with the use of excessive amount of free RGDK, is intended to mask RGDK-specific receptors, integrin αvβ3/5 and neuropilin-1, on cells to hamper RGDK-related cellular uptake. In Figure 2, the uptake of HFn-GFLG/PLGLAG-PAS-RGDK groups were significantly inhibited by RGDK peptide pre-incubation while in other groups no obvious difference occurred. After the pre-incubation of RGDK peptide, internalized fluorescence intensities of HFn-GFLG-PAS-RGDK/DOX and HFn-PLGLAG-PAS-RGDK/DOX were similar to that of HFn/DOX and HFn-PAS/DOX. This proves that RGDK facilitated 4T1 cells’ internalization of HFn-GFLG-PAS-RGDK/DOX and HFn-PLGLAG-PAS-RGDK/DOX by providing RGDK-specific receptor-mediated pathway.

The difference of tumor cell uptake efficiencies lies in various uptake mechanisms. DOX is a small molecule and enters cells via passive diffusion. Passive diffusion is energy-free and concentration gradient-driven. It is quicker compared with all other internalization pathways when directly incubating drugs with cells. As 4T1 does not express human TFR1, HFn/DOX and HFn-PAS/DOX probably enter the cell through non-specific pinocytosis. In contrast with HFn/DOX and HFn-PAS/DOX, HFn-GFLG-PAS-RGDK/DOX and HFn-PLGLAG-PAS-RGDK/DOX have an extra internalization pathway by binding to RGDK recognized receptors, integrin αvβ3/5 and neuropilin-1.

### 3.3. Intracellular Distribution

**Figure 3:**
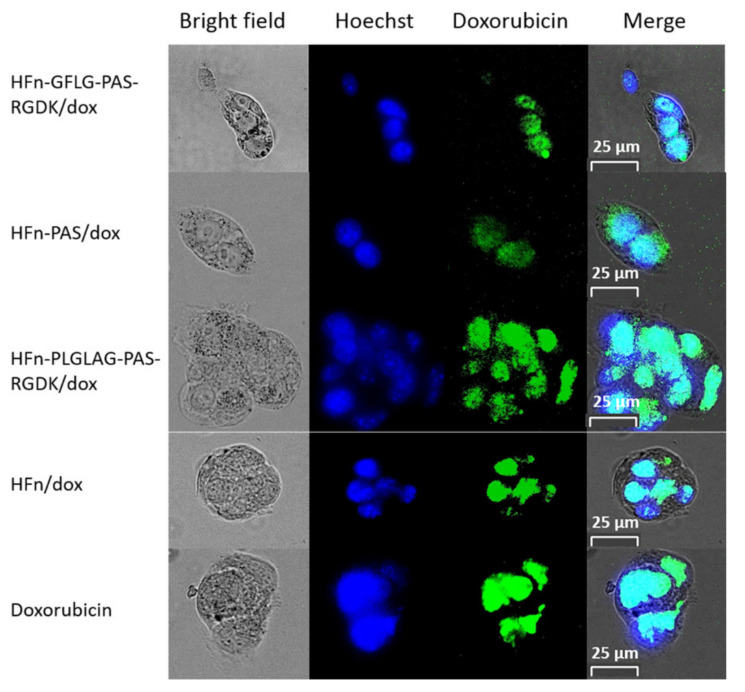
Intracellular distribution of DOX in 4T1 cells. Under the Bio-Rad Zoe cell imager, blue: nucleus after being stained with Hoechst 33258. Green: DOX because of its intrinsic fluorescence. Cyan: merged florescence signal.

DOX is an anthracycline topoisomerase inhibitor and exerts its function mainly inside the cell nucleus [24]. Free DOX directly diffuses into nucleus and disrupts cell division after internalization, whilst the protein/DOX are supposed to first be broken down by enzymes in lysosome to release loaded DOX and then reach nucleus. Intracellular distribution test was designed to check if the drugs loaded on HFn-based proteins could enter cell nucleus to kill tumor cells. In Figure 3, the blue color indicates where cell nucleus is and green color represents the fluorescence from DOX. Clearly, the majority of DOX has entered and accumulated inside nucleus of 4T1 cells in all groups, as the cyan color is the dominant color in merged images. This shows that the DOX in four protein/DOX groups could accumulate in 4T1 cell nucleus, the same as free DOX. 

### 3.4. Functionalization Effect on Cytotoxicity

**Figure 4:**
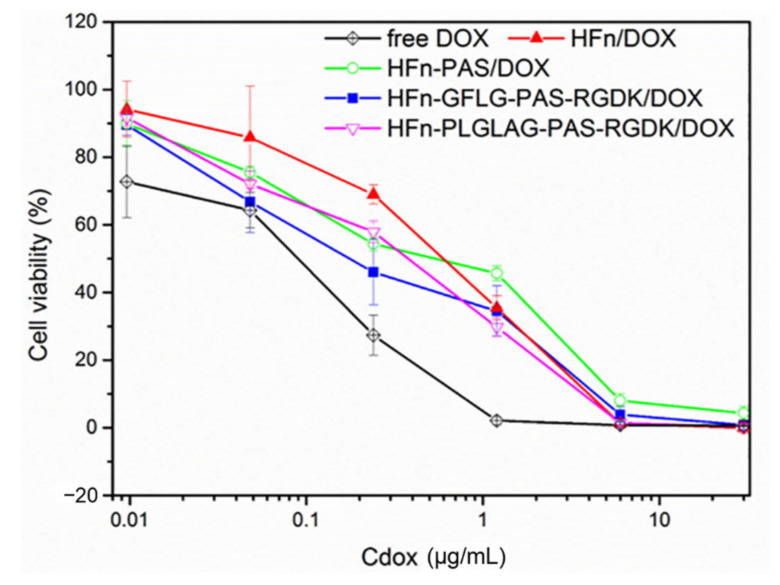
Proliferation inhibition on 4T1 cells. Data were mean ± standard deviation (*n* = 4).

In order to test the inhibition of protein/DOX on tumor cell proliferation, we adopted an MTT assay. All DOX loaded HFn-based proteins demonstrate obvious anti-proliferation abilities (Figure 4). Free DOX group had the lowest IC_50_ (Table 2) and this is due to its relatively high cellular internalization efficiency. Inhibition impacts of HFn-GFLG-PAS-RGDK/DOX and HFn-PLGLAG-PAS-RGDK/DOX on tumor cell growth were similar and the second strongest, HFn-PAS/DOX ranked third, and HFn/DOX showed the worst anti-proliferation effect. T-test shows there was significant differences between IC_50_ values of free DOX and HFn-GFLG/PLGLAG-PAS-RGDK/DOX group (*p* < 0.05). Significant differences of IC_50_ values were also found between HFn-GFLG/PLGLAG-PAS-RGDK/DOX and the other two HFn-based protein/DOX groups (*p* < 0.05). That implies the RGDK in HFn-GFLG/PLGLAG-PAS-RGDK/DOX has enhanced HFn/DOX performance in terms of drug cytotoxicity towards tumor cells.

### 3.5. Functionalization Effect on Pharmacokinetic Profile 

**Figure 5:**
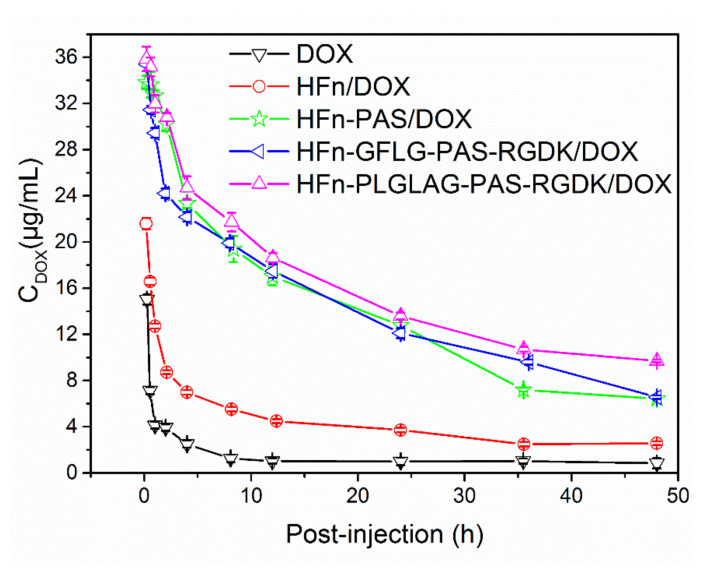
Plasma concentrations of protein/DOX and free DOX in Sprague Dawley rats of different groups. Data were expressed as mean ± SD (*n* = 3).

Pharmacokinetic profile of all four protein/DOX and free DOX were obtained through tail vein injection of healthy Sprague Dawley rats. Line chart of DOX concentrations in plasma over time (10 min–48 h) is shown in Figure 5 and half-lives in circulation of all protein/DOX are listed in Table 3. Standard curve of fluorescence intensity-doxorubicin concentration is shown in supplementary material Figure S1. Plasma drug concentrations of free DOX group rats reduced rapidly right after administration (Figure 5). At 10 min, average plasma drug concentration was 15 µg mL^−1^. Then, 8 h later, almost all free DOX was eliminated from circulation. In terms of HFn/DOX group rats, their plasma drug cleaning out speed ranked the second, with average drug concentration of 21.6 µg mL^−1^ at 10 min and below 5 µg mL^−1^ after 12 h. Average plasma drug concentrations in all three functionalized HFn/DOX group rats (approximate 35 µg mL^−1^) were more than double of those in free DOX group at 10 min. 48 h after administration, more than 5 µg mL^−1^ drug still remained in plasma of all three functionalized HFn/DOX group rats. Single compartment fitting of the drug concentration-time curve was applied to evaluate drug half-life in circulation. The results show free doxorubicin only had about 25 min of half–life in circulation (Table 3). HFn/DOX half-life, approximate 3 h, was 7.3 times of free DOX. PAS peptide in HFn-PAS/DOX has increased half-life almost 4.9 times (14.96 h) compared with HFn/DOX. The extra RGDK residues in HFn-GFLG-PAS-RGDK/DOX further extended the half-life to 17.61 h. HFn-PLALGA-PAS-RGDK/DOX possessed the longest half-life, 18.93 h. Differences in half-life of all three functionalized HFns/DOX compared with HFn/DOX and free drug were statistically significant in *t*-test (*p* < 0.001). 

### 3.6. Functionalization Effect on Protein Biodistribution

To monitor distribution of all HFn-based proteins in tumor-bearing mice after tail vein administration over time, we used in vivo imaging. In this analysis, fluorescence label Cy5 was attached to all employed proteins and free cy5 worked as free drug control. The reagent in use reacts with primary amine group on protein outer surface. On the outer surface of HFn assembly, there are 24 exposed subunit N-terminals –NH_2_ groups and 144 Lys (K) residues. Due to the large number of accessible reaction sites, the possibility of cy5 blocking some or all K residues of RGDK in HFn-GFLG/PLGLAG-PAS-RGDK is low. The cy5 conjugation is unlikely to affect RGDK function. Real-time biodistribution of Cy5 attached proteins and free Cy5 were visualized in BALB/c mice with 4T1 tumor in right armpit, and fluorescence intensities of tumor areas were recorded. Two control groups, mice injected with free cy5 and HFn-cy5 were scanned at the same time, and mice in other three groups (HFn-PAS-cy5, HFn-PLALGA-PAS-RGDK-cy5 and HFn-GFLG-PAS-RGDK-cy5) were scanned together. 

**Figure 6:**
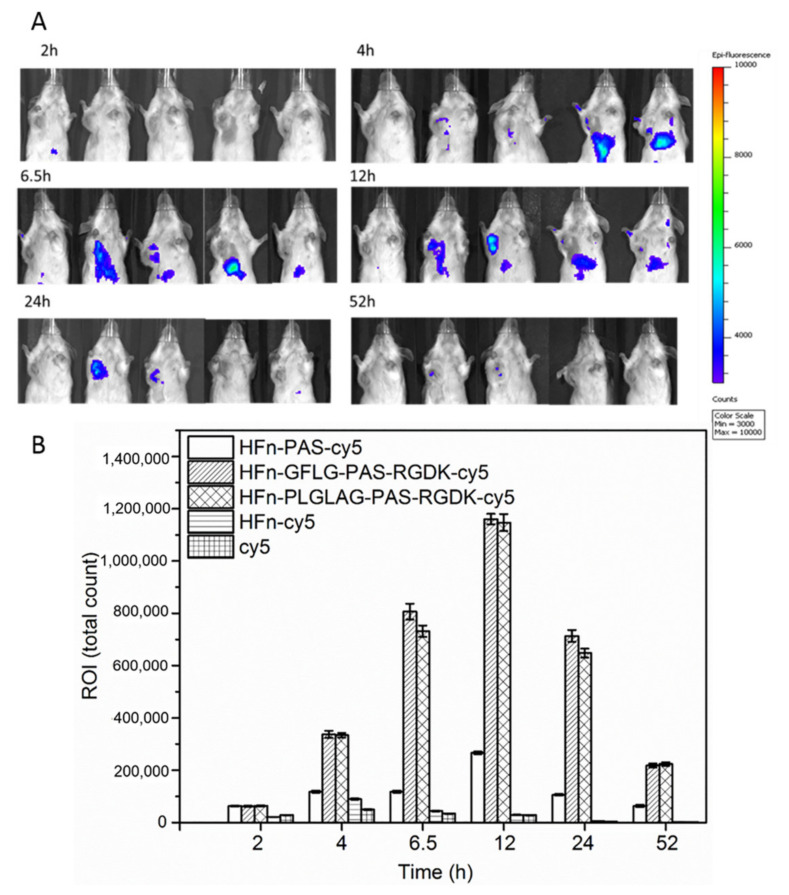
Biodistribution of cy5 and cy5 conjugated with HFn-based proteins. (**A**), in vivo fluorescence imaging of tumor-bearing mice at different time points, from left to right: HFn-PAS-cy5, HFn-GFLG-PAS-RGDK-cy5, HFn-PLGLAG-PAS-RGDK-cy5, HFn-cy5 and free cy5. (**B**), the sum fluorescent intensity of region of interest (ROI, tumor area) at each time point.

Figure 6A shows the top half of the mice where there was fluorescence signal captured by camera. 4–12 h after injection, fluorescence of free cy5 in liver was captured by camera (Figure 6A). At 24 and 52 h, fluorescence was barely visible. Free cy5 preferred to accumulate in the liver, perhaps due to the fact that liver is the main organ for metabolism. Theoretically, as a nanoparticle, HFn has passive tumor targeting ability. However, from the results in Figure 6A, signal of HFn-cy5 fluorescence was captured in liver rather than in tumor from 4 to 12 h. It seems that HFn-cy5 did not show desirable tumor targeting ability and it preferred liver. The particle size of HFn is probably still too small to achieve desirable passive tumor targeting ability. No obvious fluorescence was captured at all time points in HFn-PAS-cy5 group (Figure 6A). However, as is shown in Figure 6B, there actually was fluorescence detected in tumor area. Perhaps because of the sharp contrast between signal intensities of HFn-PAS-cy5 and HFn-GFLG/PLGLAG-PAS-RGDK-cy5, lower intensity of HFn-PAS-cy5 failed to be captured by the camera under the same exposure time. In HFn-GFLG/PLGLAG-PAS-RGDK-cy5 groups, fluorescence signal was captured from 4 h to 52 h after injection (Figure 6A). The armpit fluorescence areas at 6.5, 12 and 24 h were larger than the area of armpit lymph node, proving the protein accumulation in tumor tissues. However, it is uncertain that if lymph node accumulation co-existed or not. Figure 6A shows that the tumor area of HFn-GFLG/PLGLAG-PAS-RGDK-cy5 groups had stronger signals than liver at all time points. At 52 h after injection, whilst HFn-cy5 and free cy5 were almost completely eliminated, HFn-GFLG/PLGLAG-PAS-RGDK-cy5 were still detectable in region of tumor site, implying functionalized HFns were retained in tumor by longer and stronger accumulation.

As is presented in Figure 6B, free cy5 and HFn-cy5 had the lowest tumor florescence intensity at all time points. At 2 h, free cy5 had a greater intensity than HFn-cy5 but was surpassed by HFn-cy5 afterwards. Free cy5 tumor area fluorescence intensity peaked at 4 h and decreased rapidly after that, suggesting a fast clearance. HFn-cy5 achieved the highest concentration in tumor at around 4 h after injection (Figure 6B). The difference in free cy5 and HFn-cy5 is likely to be due to a quicker distribution and a shorter half-life of small molecule cy5 than HFn-cy5. HFn-PAS-cy5 demonstrated significantly stronger and longer lasting tumor intensities than HFn-cy5 at all detected time points (*p* < 0.001). As proven in the pharmacokinetic study, the insertion of the PAS peptide could lead to a longer half-life in circulation and probably result in the slower clearance of HFn-PAS-cy5 than HFn-cy5. The best drug targeting delivery results were from HFn-GFLG/PLGLAG-PAS-RGDK-cy5. They had significantly greater signal intensities in tumor area at all times than all the other groups (*p* < 0.001). This shows that the RGDK peptide can significantly improve HFn biodistribution. A previous study of RGDK fused Albumin binding domain has also proven the tumor targeting ability improvement of RGDK peptide *in vivo*. [24] Overall, both PAS and RGDK functionalization, and particularly RGDK functionalization, improved the tumor biodistribution of HFn.

### 3.7. Functionalization Effect on Protein/DOX Anti-Tumor Efficacy

**Figure 7:**
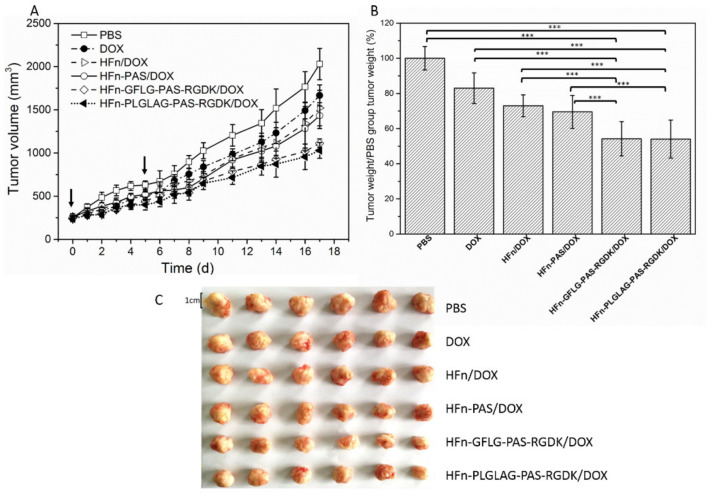
In vivo tumor inhibitory effects on 4T1 tumor-bearing mice. (**A**), tumor volume change over time. (**B**), group tumor weight/ PBS group tumor weight (%) on day 17. *** *p* < 0.001. Symbol ‘*’ represents the significance of *p* value between groups. (**C**), the photo of excised tumor tissues on day 17. Arrows indicated the injection days; data are mean ± RSD (*n* = 6).

To compare tumor treatment efficacies of all protein/DOX and free DOX, 4T1 tumor bearing BALB/c mice model was built and 36 mice with around 250 mm^3^ tumor were randomly assigned into six groups. Intravenous injections of four HFn-based protein/DOX, free DOX and PBS were conducted at day 0 and day 5. As is shown in Figure 7A, the fastest mice tumor growth rate was observed in PBS control group rats which underwent no drug treatment. The average tumor volume reached 2030 mm^3^ after 17 days. The second fastest tumor growth rate was in free DOX group mice and their average group tumor volume were 1667 mm^3^ at day 17. HFn/DOX showed a better tumor growth inhibition and at day 17, tumor volume grew to 1521 mm^3^. In HFn-PAS/DOX group, mice tumor volume reached 1432 mm^3^ in the end. Two PAS-RGDK functionalized HFn/DOX treated group had the strongest tumor-growth inhibition. In spite of just two administrations, average tumor volume of these two group mice at day 17 were just around 1100 mm^3^, close to half of the volume of the PBS group tumor.

On day 17, the corresponding average tumor weights were measured and the percentages of tumor weights compared with tumor weights of control PBS group mice are presented in Figure 7B. The photo of excised tumor tissues is shown as Figure 7C. Tumor weights on day 17 of HFn-GFLG-PAS-RGDK/DOX and HFn-PLGLAG-PAS-RGDK group mice were 54.2 ± 9.7% and 54.0 ± 10.8% of tumor weights of the control PBS group. HFn-PAS/DOX group mice had 69.5 ± 9.4% and HFn/DOX group mice had 72.99 ± 6.2% of PBS control group mice tumor weights. Free DOX group mice tumor weight was 82.9 ± 8.7% of PBS control group mice. T-test results demonstrate that there were significant statistical differences between final tumor masses of protein/DOX and free DOX group. Both HFn and functionalized HFns had significantly increased DOX anti-tumor efficacy (*p* < 0.001). Compared with HFn/DOX group, HFn-PAS/DOX did not show statistical distinction (*p* = 0.468), showing that PAS functionalization alone was not enough to significantly improve anti-tumor efficacy. Masses of tumors from two PAS-RGDK protein/DOX groups, however, were significantly lower than those of both HFn/DOX group and HFn-PAS/DOX group. This indicates RGDK functionalization primarily accounts for the significant improvement of growth inhibition efficacy of 4T1 tumor. Difference between HFn-GFLG-PAS-RGDK/DOX and HFn-PLGLAG-PAS-RGDK/DOX (*p* = 0.977) was not significant in statistical analysis. Two different enzyme-cleavable sites did not make a statistical difference in anti-tumor efficacy.

## 4. Discussion

Based on all of the results above, PAS and RGDK functionalization have both improved HFn anti-tumor performance. PAS functionalization impacts HFn mainly by extension of half-life in circulation. In pharmacokinetic study, differences in half-lives in circulation of all protein/DOX mostly stemmed from the insertion of PAS peptide. In biodistribution assay, PAS provided HFn-PAS with a longer retaining time than HFn in tumor area. Constituted by repetitive P, A and S residues, PAS peptide is hydrophilic and uncharged in neutral solutions and plasma. Circular dichroism shows it is a flexible random coil [25]. Its properties are similar to polyethylene glycol (PEG), but it is advantageous in terms of biodegradability and biocompatibility. When it is attached to another molecule, it can attract water molecules to increase molecule hydrodynamic volume, thereby extending half-life in circulation. PAS peptide usually has to be over 200 residues to achieve half-life extension, but because of the repeated and organized presentation manner on ferritin shell, 40 aa and 75 aa PAS peptides have been proven to be long enough when fused onto N-terminal of ferritin subunit [11]. In this study, the enlargement of HFn hydrodynamic volume after PAS insertion was detected in HPSEC-MALLS characterization. 

RGDK peptide significantly improved anti-tumor performance of HFn/DOX. In cellular uptake assay, it has increased cellular internalization efficiency through binding to specific receptors. It has also led to the best tumor targeting abilities in biodistribution assay and the greatest anti-tumor efficacy in cytotoxicity and in vivo anti-tumor assay.

Comparing HFn-GFLG-PAS-RGDK and HFn-PLGLAG-PAS-RGDK, the sequence length difference caused the minor differences in hydrodynamic volume in HPSEC-MALLS and half-life in circulation in pharmacokinetic study. However, the two enzyme-cleavable sites, GFLG and PLGLAG, did not make a significant difference in cellular uptake efficiency, and in any other in vitro and in vivo tests. This suggest the PLGLAG enzyme-cleavable site probably was not digested by MMP-2/9 before cell internalization. It could be caused by the insufficient activity of MMP-2/9 in vitro and in vivo and/or the low accessibility of PLGLAG to enzymes. A further detailed investigation of the cleavage of PLGLAG is needed. 

**Figure 8:**
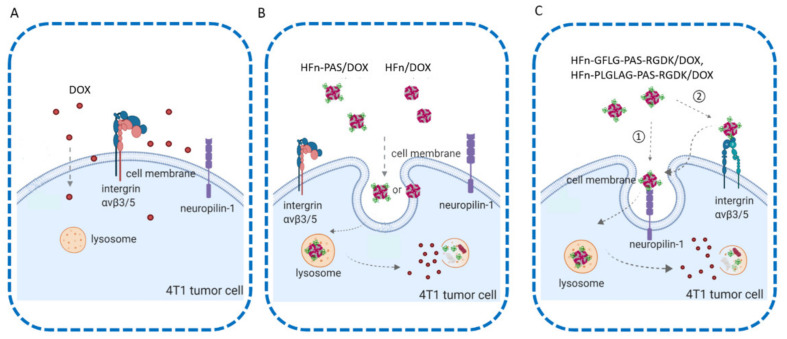
Schematic of different tumor cellular internalization mechanisms of DOX and protein/DOX. (**A**), free DOX passive diffusion pathway. (**B**), HFn/DOX and HFn-PAS/DOX pinocytosis internalization. (**C**), two possible receptor-mediated internalization pathways of HFn-GFLG-PAS-RGDK/DOX and HFn-PLGLAG-PAS-RGDK. (Created with Biorender).

Figure 8 illustrates the assumed tumor cell internalization pathways of all groups. Free DOX enters tumor cells via unspecific passive diffusion due to its small size (Figure 8A). The short half-life in circulation and the lack of tumor targeting ability caused a great loss of DOX before it reached tumor cells. As a result, the in vivo anti-tumor efficacy was the lowest. Both HFn/DOX and HFn-PAS/DOX enter cells through non-specific pinocytosis [26] (Figure 8B), because there is no corresponding receptor, human TfR1, on 4T1 cells. Therefore, in in vitro assessments, cellular uptake assay and cytotoxicity assay, there were no statistical differences between HFn/DOX and HFn-PAS/DOX. 

HFn-GFLG-PAS-RGDK/DOX and HFn-PLGLAG-PAS-RGDK/DOX have extra drug internalization mechanisms compared with HFn/DOX and HFn-PAS/DOX (Figure 8C). Since RGDK has RGD motif and an exposed free C-terminal K residue, it can be directly recognized by both integrin αvβ3/5 and neuropilin-1 (NRP1), two kinds of receptors overexpressed on 4T1 cells [14]. The overexpression of these two receptors has led to the increase of internalization efficiency of HFn-GFLG/PLGLAG-PAS-RGDK/DOX. As shown in Figure 8C, when HFn-GFLG/PLGLAG-PAS-RGDK/DOX reaches tumor tissue, there are two possible internalization pathways. RGDK can either directly bind to NRP1 or firstly interact with integrin αvβ3/5 and then be transferred to NRP1, followed by endocytosis. After that, some of the HFn-GFLG/PLGLAG-PAS-RGDK/DOX inside tumor cells would be digested in lysosome while some will travel to other cells nearby via paracellular pathway or transcytosis [27].

## 5. Conclusions

All three functionalized HFns expressed in *E. coli* have self-assembled into nanoparticles such as HFn. RGDK peptide has enhanced HFn tumor cell uptake efficiency and improved biodistribution, resulting in a significant improvement in anti-tumor treatment outcome. PAS has expanded HFn hydrodynamic volume and helped ferritin stay longer in circulation, which also has improved anti-tumor efficacy of ferritin. In summary, we successfully prepared and evaluated three new functionalized HFn constructs (HFn-PAS, HFn-GFLG-PAS-RGDK, HFn-PLGLAG-PAS-RGDK), especially two PAS-RGDK fused ones, which hold greater potentials as anti-tumor drug delivery nanoparticles than HFn.

## Figures and Tables

**Table 1:** Hydrodynamic radius and molecular weight determined by HPSEC-MALLS.

Protein	Particle Hydrodynamic Radius (nm)	Measured Average Molecular Weight (kDa)	Theoretical Molecular Weight (kDa)
HFn	6.31 (±0.53%)	493.3(±0.07%)	506.0
HFn-PAS	7.72 (±0.54%)	608.6(±2.18%)	625.1
HFn-GFLG-PAS-RGDK	8.06 (±0.55%)	630.8(±0.12%)	636.1
HFn-PLGLAG-PAS-RGDK	8.22 (±0.55%)	638.7(±0.11%)	639.3

**Table 2:** IC_50_ values of all groups.

Group	IC_50_ (μg mL^−1^)
DOX	0.08 ± 0.03
HFn/DOX	0.49 ± 0.11
HFn-PAS/DOX	0.38 ± 0.09
HFn-GFLG-PAS-RGDK/DOX	0.17 ± 0.01
HFn-PLGLAG-PAS-RGDK/DOX	0.18 ± 0.04

**Table 3:** Half-life of each protein/DOX in Sprague Dawley rats (*n* = 3).

Group	T_1/2_(h)
DOX	0.42 ± 0.03
HFn/DOX	3.07 ± 0.06
HFn-PAS/DOX	14.96 ± 0.29
HFn-GFLG-PAS-RGDK/DOX	17.61 ± 0.39
HFn-PLGLAG-PAS-RGDK/DOX	18.93 ± 0.61

## Data Availability

The data presented in this study are available on request from the corresponding author.

## Supplementary Materials

The following are available online at https://www.mdpi.com/article/10.3390/pharmaceutics13040521/s1, Figure S1: standard curve of fluorescence intensity-doxorubicin concentration in SD rat plasma.
