# Supplementary material for: Engineered Human Heavy-Chain Ferritin with Half-Life Extension and Tumor Targeting by PAS and RGDK Peptide Functionalization
Source: Pharmaceutics. 2021 Apr 9;13(4):521. doi: 10.3390/pharmaceutics13040521 (PMC8070472; doi:10.3390/pharmaceutics13040521)
Supplement: Supplementary file 1 [file pharmaceutics-13-00521-s001.pdf]

pharmaceutics

Supplementary Materials: Engineered Human Heavy-Chain Ferritin with Half-Life Extension and Tumor Targeting by PAS and RGDK Peptide Functionalization

Shuang Yin, Yan Wang, Bingyang Zhang, Yiran Qu, Yongdong Liu, Sheng Dai, Yao Zhang, Yingli Wang and Jingxiu Bi

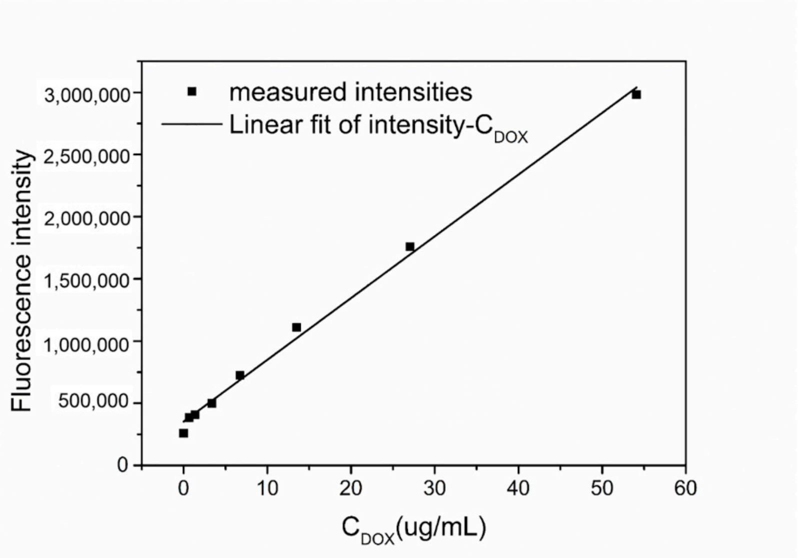

Figure S1. Standard curve of fluorescence intensity-doxorubicin concentration in SD rat plasma. Fluorescence intensity = 49656 C_{DOX} +353005, R2 = 0.996.
